# Ticagrelor vs. clopidogrel for coronary microvascular dysfunction in patients with STEMI: a meta-analysis of randomized controlled trials

**DOI:** 10.3389/fcvm.2023.1102717

**Published:** 2023-05-18

**Authors:** Yike Li, Zixiang Ye, Ziyu Guo, Enmin Xie, Min Wang, Xuecheng Zhao, Mei Liu, Peizhao Li, Changan Yu, Yanxiang Gao, Jingang Zheng

**Affiliations:** ^1^ Department of Cardiology, China-Japan Friendship Hospital (Institute of Clinical Medical Sciences), Chinese Academy of Medical Sciences and Peking Union Medical College, Beijing, China; ^2^ Department of Cardiology, Peking University China-Japan Friendship School of Clinical Medicine, Beijing, China; ^3^ Department of Cardiology, China-Japan Friendship Hospital, Beijing, China

**Keywords:** ST-elevation myocardial infarction, ticagrelor, clopidogrel, coronary microcirculation dysfunction, meta-analysis

## Abstract

**Purpose:**

Approximately half of ST-segment elevation myocardial infarction (STEMI) patients who undergo revascularization present with coronary microvascular dysfunction. Dual antiplatelet therapy, consisting of aspirin and a P2Y12 inhibitor (e.g., clopidogrel or ticagrelor), is recommended to reduce rates of cardiovascular events after STEMI. The present study performed a pooled analysis of randomized controlled trials (RCTs) to compare effects of ticagrelor and clopidogrel on coronary microcirculation dysfunction in STEMI patients who underwent the primary percutaneous coronary intervention.

**Methods:**

The PubMed, Embase, Cochrane Library, and Web of Science databases were searched for eligible RCTs up to September 2022, with no language restriction. Coronary microcirculation indicators included the corrected thrombolysis in myocardial infarction (TIMI) frame count (cTFC), myocardial blush grade (MBG), TIMI myocardial perfusion grade (TMPG), coronary flow reserve (CFR), and index of microcirculatory resistance (IMR).

**Results:**

Seven RCTs that included a total of 957 patients (476 who were treated with ticagrelor and 481 who were treated with clopidogrel) were included. Compared with clopidogrel, ticagrelor better accelerated microcirculation blood flow [cTFC = −2.40, 95% confidence interval (CI): −3.38 to −1.41, *p *< 0.001] and improved myocardial perfusion [MBG = 3, odds ratio (OR) = 1.99, 95% CI: 1.35 to 2.93, *p *< 0.001; MBG ≥ 2, OR = 2.57, 95% CI: 1.61 to 4.12, *p *< 0.001].

**Conclusions:**

Ticagrelor has more benefits for coronary microcirculation than clopidogrel in STEMI patients who undergo the primary percutaneous coronary intervention. However, recommendations for which P2Y12 receptor inhibitor should be used in STEMI patients should be provided according to results of studies that investigate clinical outcomes.

## Introduction

According to the World Health Organization, ischemic heart disease remained the leading cause of death globally in 2019, and myocardial infarction (MI) was a major threat (1). The primary percutaneous coronary intervention (PCI) is the dominant reperfusion strategy for ST-segment elevation myocardial infarction (STEMI) patients (2). Although STEMI patients received timely reperfusion therapy and the recommended drugs, the 6-month mortality rate still reached 5.3% in 2015 (3). Al-Lamee et al. (4) reported that ∼32% of STEMI patients who did not receive myocardial perfusion after PCI had poorer prognoses than patients who received complete reperfusion. The no-flow phenomenon is described as the inability to reperfuse a region of the myocardium despite the reopening of an infarct-related artery and is an independent predictor of death and MI (5). Coronary microcirculation is known to provide 95% of blood flow resistance, suggesting that coronary microcirculation is crucial in myocardial perfusion (6). Some studies suggested that the no-flow phenomenon is related to microvascular obstruction, spasm, microthrombotic embolization, and reperfusion injury (5, 7). Furthermore, some studies found that platelets play an important role in poor reperfusion, including platelet aggregation, the formation of microthrombi in microvessels, and the release of vasoconstrictors, such as thromboxane A2 (8).

Antiplatelet therapy is the principal treatment strategy for acute coronary syndrome (ACS), in addition to timely revascularization (9). The P2Y12 receptor inhibitors ticagrelor and clopidogrel are recommended to decrease the primary composite outcome of death from cardiovascular causes, nonfatal MI, and stroke (10). Several experimental studies recently suggested that ticagrelor is superior to clopidogrel in patients post-PCI in terms of improving microvascular function. However, other trials found no difference between ticagrelor and clopidogrel (11, 12). Therefore, we conducted an analysis of randomized controlled trials (RCTs) and compared improvements in coronary microcirculation in STEMI patients who were treated with ticagrelor *vs*. clopidogrel.

## Methods

We conducted a meta-analysis according to the Preferred Reporting Items for Systematic Reviews and Meta-Analyses (PRISMA) statement (13). The analysis plan was registered with the International Prospective Register of Systematic Reviews (PROSPERO, CRD42021284263).

### Data sources and search strategy

We searched for all published RCTs in the PubMed, Embase, Web of Science, and Cochrane Library databases from the databases' inception to September 2022. To ensure all relevant studies were included, we used combinations of the following keywords: *ticagrelor*, *clopidogrel*, *microcirculation*, *microvascular*, *blood flow*, *coronary flow*, *myocardial perfusion*, *coronary circulation*, and *reperfusion*. We placed no restrictions on published year, language, or article type. The detailed search strategy is shown in Supplementary Table S1. Two authors performed the study selection independently, and a third author resolved any disagreements.

### Inclusion and exclusion criteria

We included trials in the meta-analysis when they met the following criteria: (a) the study was an RCT, (b) ticagrelor and clopidogrel treatment were both administered, and the medications were compared with each other, (c) individuals who were studied were diagnosed with STEMI and treated with PCI, and (d) the publication reported information about endpoints that are associated with microcirculation, measured *via* invasive methods, including corrected thrombolysis in myocardial infarction (TIMI) frame count (cTFC), myocardial blush grade (MBG), TIMI myocardial perfusion grade (TMPG), coronary flow reserve (CFR), and index of microcirculatory resistance (IMR). We excluded reviews, meta-analyses, meeting abstracts, nonclinical studies, case reports, and duplicate literature. If indicators of microcirculation were reported in a single study but did not exist in other studies, then we removed the single study.

### Data extraction and quality assessment

Two authors independently reviewed the eligibility and methodological quality of each study using standardized data abstraction forms. A third author resolved disagreements. The following data were extracted: (a) basic information about the trial (i.e., author name, publication year, sample size, and intervention measures) and (b) baseline comorbidities, medication dose, and indicators of microcirculation.

### Statistical analysis

The *I*^2^ test was used to evaluate heterogeneity. The random-effect model would be used if significant heterogeneity is considered. Apparent clinical heterogeneity was processed by a subgroup analysis, sensitivity analysis, or descriptive analysis alone. A fixed-effect model was used when no significant heterogeneity was found. A random-effect model was then used to test sensitivity. The pooled effects of continuous variables are reported as the mean difference (SMD) and 95% confidence intervals (CIs). Dichotomous variables were estimated with odds ratios (ORs) and 95% CIs. We investigated publication bias use Egger's test (14). We considered results with *p *< 0.05 as statistically significant. Further, trim and fill analyses were performed to detect the reliability of our estimate by detecting potential missing studies due to publication bias and recalculate the pooled prevalence by taking those missing studies into account (15). The statistical analyses were performed using STATA 14.0 software (Stata Corporation, Texas, USA).

## Results

### Literature search results

**Figure 1:**
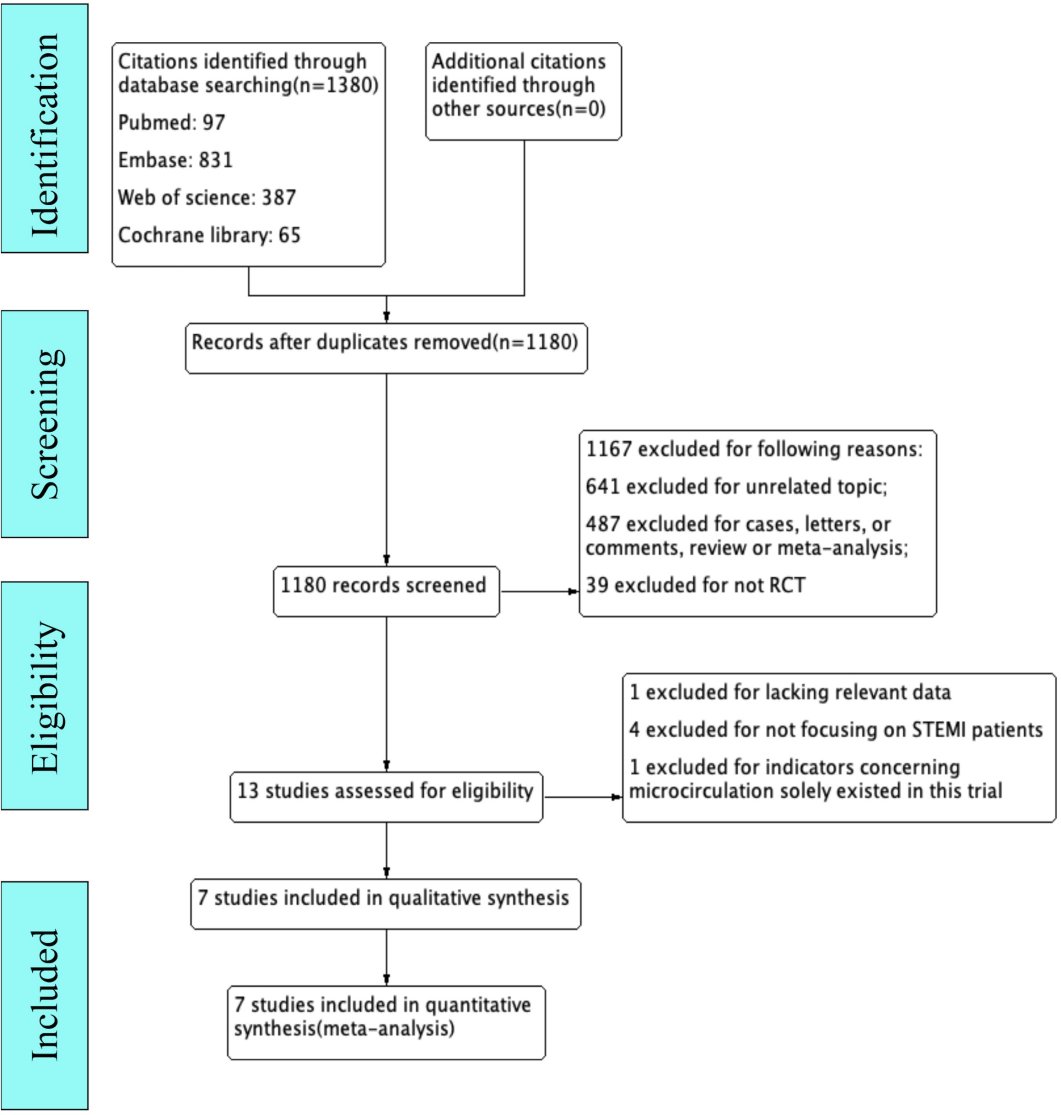
Flowchart of study selection.

Initially, 1,380 records were found based on our search strategy, 200 of which were duplicates. After screening titles and abstracts, we removed 1,167 records based on the inclusion and exclusion criteria and retrieved 13 RCTs for full-text review (Figure 1). Five publications were eliminated (one article investigated the ACS population but lacked detailed data on STEMI patients; four trials did not focus on STEMI patients; in one trial, indicators of microcirculation did not appear in other studies). We finally included seven RCTs (16–22) in the present analysis.

### Study characteristics and quality assessment

The seven RCTs involved a total of 957 STEMI subjects that were included in the present analysis, of which 476 were assigned to ticagrelor and 481 were assigned to clopidogrel. Tables 1, 2 list the study characteristics and baseline patient demographics. The patients' mean ages ranged from 55.1 to 69.1 years. Most patients were male and had hypertension and a history of smoking. A smaller proportion of the participants, with the exception of the study by Liu et al. (21), had diabetes mellitus. There was a similar proportion of patients with dyslipidemia in both groups, with the exception of the studies by Winter et al. (16) and Mont'Alverne-Filho et al. (17). In these trials, loading doses of ticagrelor and clopidogrel were administered after the diagnosis of STEMI, and coronary microcirculation indices were calculated by analyzing coronary angiography images after PCI. Among these trials, five reported microvascular perfusion with cTFC and MBG, but one study of MBG did not report completely, and another lacked concrete data.

**Table 1:** Major characteristics of included trials.

Trial	Year	Study design	No. of patients on ticagrelor	No. of patients on clopidogrel	Ticagrelor dosing regimen	Clopidogrel dosing regimen	Microcirculation perfusion index
Winter JL et al. (14)	2014	Single-center, prospective, randomized, open-label, blinded-endpoint study	34	36	Loading 180 mg, maintenance 90 mg twice daily	Loading 600 mg, maintenance 75 mg daily	cTFC MBG
Mont’Alverne-Filho JR et al. (15)	2016	Single-center, prospective, randomized, blinded-endpoint study	46	44	Loading 180 mg	Loading 600 mg	MBG
Li WH et al. (16)	2018	Single-center, prospective, randomized, blinded-endpoint study	20	20	Loading 180 mg	Loading 600 mg	cTFC
Wang X et al. (17)	2019	Single-center, prospective, randomized, blinded-endpoint study	150	148	Loading 180 mg, maintenance 90 mg twice daily	Loading 600 mg, maintenance 75 mg daily	cTFC MBG
Cao B et al. (18)	2019	Single-center, prospective, randomized study	49	48	Loading 180 mg, maintenance 90 mg twice daily	Loading 600 mg, maintenance 75 mg daily	cTFC
Liu Y et al. (19)	2019	Single-center, prospective, randomized study	108	100	Loading 180 mg, maintenance 90 mg twice daily	Loading 600 mg, maintenance 75 mg daily	MBG
Hamilos M et al. (20)	2021	Multicenter, prospective, randomized, open-label, blinded-endpoint study	69	85	Loading 180 mg, maintenance 90 mg twice daily	Loading 300 mg, maintenance 75 mg daily	cTFC TMPG MBG

cTFC, corrected TIMI frame counts; MBG, myocardial blush grade; TMPG, TIMI myocardial perfusion grade.

**Table 2:** Baseline characteristics of included trials.

Trial	Ticagrelor and Clopidogrel							
	Patients	Age, Year (Mean ± SD)	Males, %	Hypertension, %	Diabetes, %	Dyslipidemia, %	Current smoker, %	Ejection fraction, %
Winter JL et al. (14)	34/36	55.1 ± 8.3/62.1 ± 10.5	79.0/69.0	52.0/63.0	32.0/22.0	26.0/19.0	70.0/52.0	50.4 ± 8.8/46.8 ± 8.9
Mont’Alverne-Filho JR et al. (15)	46/44	58.0/58.0	60.9/68.2	60.9/45.5	32.6/34.1	37.0/45.5	41.3/50.0	-
Li WH et al. (16)	20/20	59.5 ± 11.0/58.5 ± 16.5	65.0/60.0	55.0/50.0	35.0/40.0	-	60.0/50.0	48.5 ± 7.8/53.3 ± 6.0
Wang X et al. (17)	150/148	59.7 ± 13.0/60.9 ± 12.1	81.8/76.7	58.1/59.3	23.7/20.7	14.2/12.0	64.2/60.7	-
Cao B et al. (18)	49/48	61.6 ± 11.2/62.8 ± 11.4	61.2/60.4	-	-	-	-	49.0 ± 6.0/52.0 ± 8.0
Liu Y et al. (19)	108/100	68.3 ± 4.7/69.1 ± 5.1	53.7/58.0	51.9/48.0	100.0/100.0	31.5/36.0	48.2/46.0	52.2 ± 2.9/51.3 ± 2.4
Hamilos M et al. (20)	69/85	58.0 ± 10.0/58.0 ± 9.0	84.0/90.0	26.0/32.0	15.0/14.0	21.0/24.0	65.0/62.0	48.0 ± 8.0/47.8 ± 8.0

SD, standard deviation.

A detailed bias assessment of the studies is summarized in Supplementary Table S2. All of the studies were assessed as having low to moderate risk of bias based on the Cochrane risk-of-bias tool (Supplementary Figure S1).

### Meta-analysis results

#### cTFC

**Figure 2:**
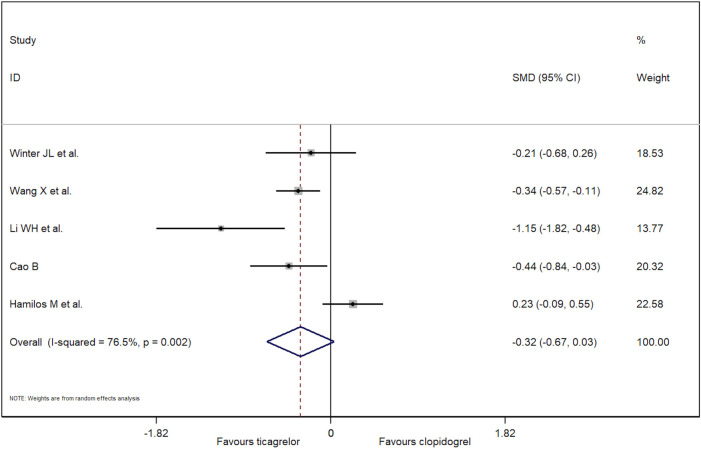
Forest plot of cTFC using random-effect model.

**Figure 3:**
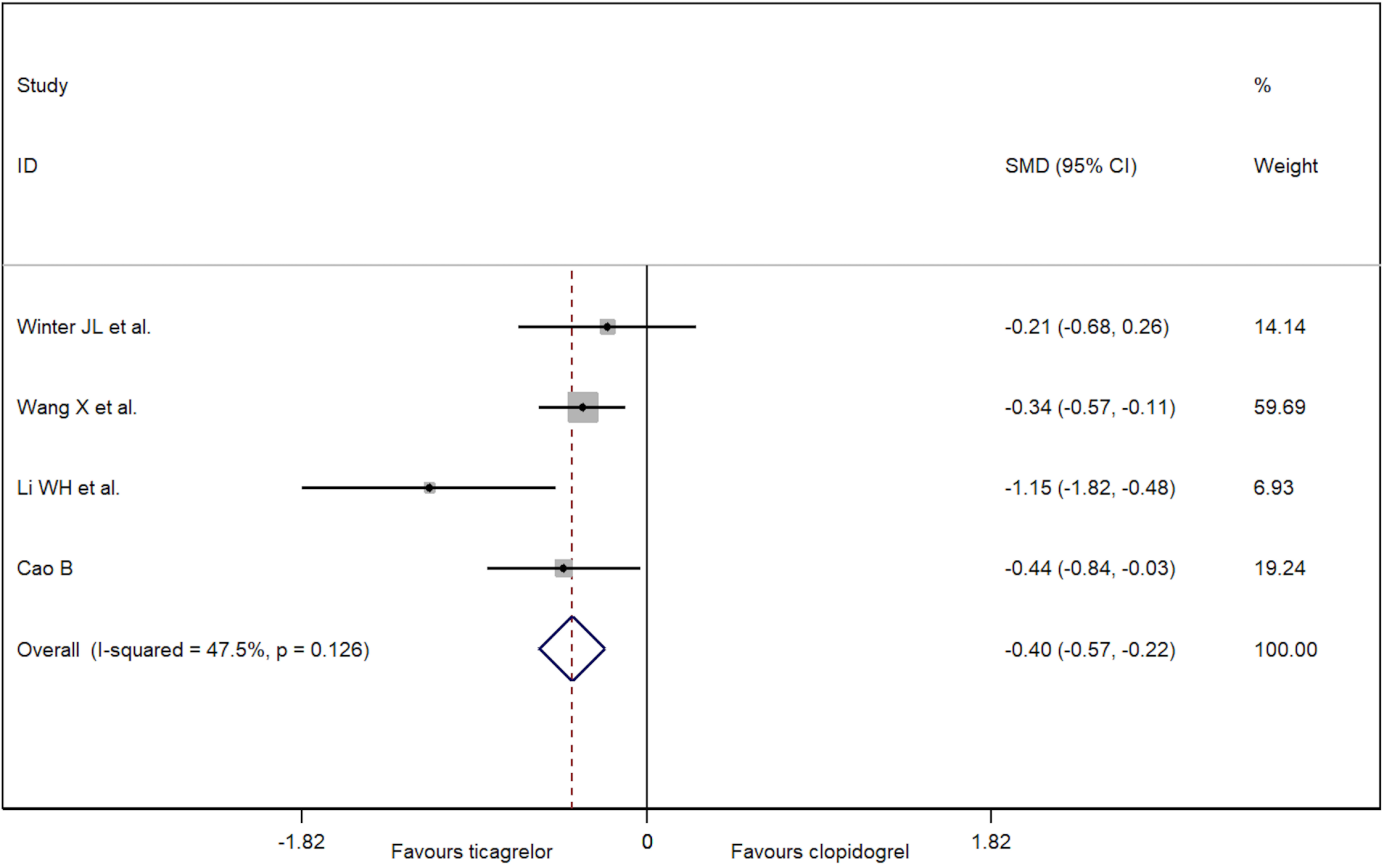
Forest plot of cTFC after removing one high-risk trial.

Five RCTs reported the outcome of cTFC. Because of significant heterogeneity, a random-effect model was used. As a result, there is no difference for cTFC between patients treated with ticagrelor and clopidogrel (−0.32, 95% CI: −0.67 to 0.03, *p *= 0.10; *I*^2 ^= 76.5%, *p *= 0.002; Figure 2). No publication bias was found (Egger's test, *p *= 0.53). A funnel plot for cTFC is shown in Supplementary Figure S2. Based on the sensitivity analysis (Supplementary Figure S3), one study had significant heterogeneity (22). In this study, patients were treated with thrombolysis before PCI, which may affect coronary microcirculation that is measured during PCI. To reduce heterogeneity and bias, we excluded this study. Compared with clopidogrel, it concluded that the patients who were treated with ticagrelor had lower cTFC (−0.40) after PCI (95% CI: −0.57 to −0.22, *p *< 0.001; *I*^2 ^= 47.5%, *p *= 0.13; Figure 3) in the random-effect model.

#### Incidence of MBG = 3

**Figure 4:**
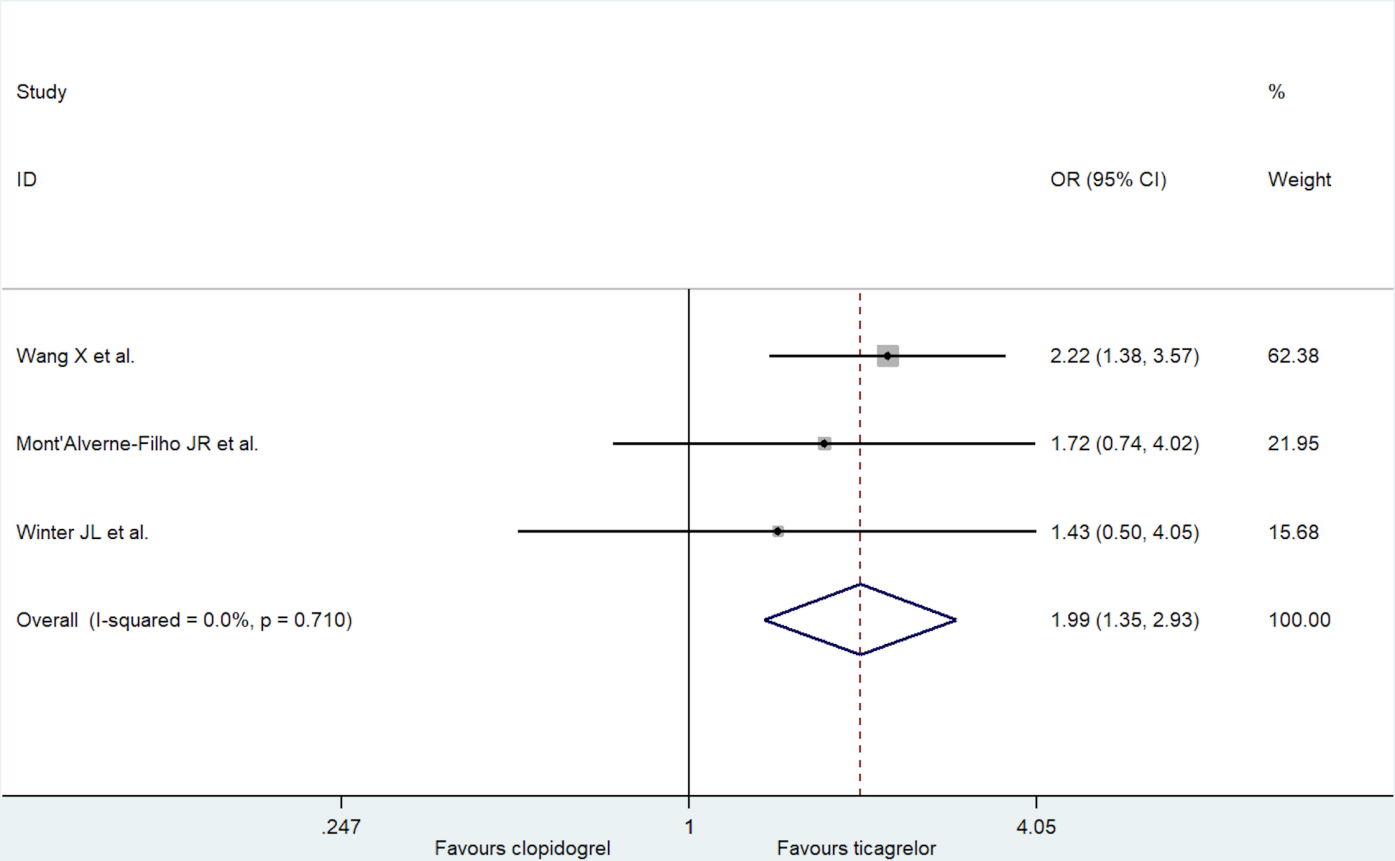
Forest plot of incidence of MBG = 3.

Three trials reported the incidence of MBG = 3. A significant publication bias was evident following the Egger's test (*p* = 0.04). Compared with clopidogrel, ticagrelor before PCI was associated with a significantly higher rate of MBG = 3 (fixed-effect model: OR = 1.99; 95% CI: 1.35 to 2.93, *p *< 0.001; *I*^2 ^= 0%, *p *= 0.71; Figure 4; and random-effect model: OR = 1.99; 95% CI: 1.35 to 2.93, *p *< 0.001). A mild increase in OR estimate was noted through trim and fill analysis (adjusted OR = 2.22; 95% CI: 1.59 to 3.11, *p *< 0.001; Supplementary Figure S4).

#### Incidence of MBG ≥2

**Figure 5:**
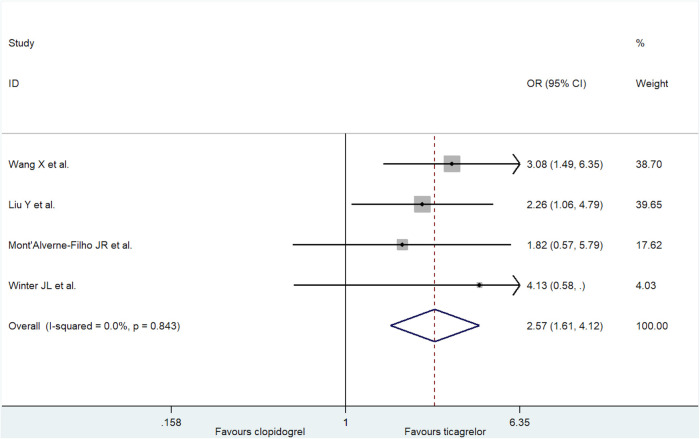
Forest plot of incidence of MBG ≥ 2.

We analyzed the incidence of MBG ≥ 2 (Figure 5). Compared with the clopidogrel group, the ticagrelor group had a greater incidence of MBG ≥ 2 (fixed-effect model: OR = 2.57; 95% CI: 1.61 to 4.12, *p *< 0.001; *I*^2 ^= 0%, *p *= 0.84; and random-effect model: OR = 2.55; 95% CI: 1.59 to 4.10, *p *< 0.001). No publication bias was found in these trials (Egger's test, *p *= 0.91).

## Discussion

The present meta-analysis of seven RCTs, including 957 STEMI patients who underwent PCI, found that ticagrelor improved microvascular perfusion more significantly than clopidogrel, measured as both cTFC and MBG.

Coronary microcirculation is defined as the vessel network that contains prearterioles (diameter <400 µm), arterioles (diameter <100 µm), and capillaries (diameter <10 µm) (23). Coronary microvascular dysfunction (CMD) can result in the inability of coronary arteries to augment coronary blood flow and can even result in a reduction of coronary blood flow, thereby contributing to ischemia in the absence of obstructive epicardial coronary artery disease (24). Impairments in coronary microvascular function play an important role in various diseases, including diabetes, chronic kidney disease, and Takotsubo syndrome (25). In STEMI patients, even if successful PCI is performed, incomplete microvascular perfusion is present in more than half of patients, along with a higher incidence of cardiac death (26). The underlying mechanisms include coronary endothelial cell injury, intramyocardial hemorrhage, greater coronary microvascular permeability, platelet aggregation, and the formation of microthrombi in microvessels (8). Coronary microcirculation resistance closely correlated with left ventricular function and myocardial infarct size in STEMI patients (27). Changes in coronary microcirculation on the first day after primary PCI are associated with the 6-month ejection fraction and myocardial salvage (28). Even after controlling for infarct size, microvascular status remained a strong predictor of prognosis in patients with MI (29).

Platelet activation can lead to the formation of thrombi and distal microemboli and obstructive platelet aggregates within myocardial capillaries in reperfused ischemic tissue (30–32). Antiplatelet treatment has been proven to reduce intracoronary thrombus and improve the speed and efficacy of epicardial reperfusion (33). Unlike clopidogrel, ticagrelor is a direct-acting, reversible antagonist of P2Y12 receptors and has a faster onset (peak activity within 30 min) and shorter half-life (8–12 h) than clopidogrel (10). In addition to its potent inhibition of platelet function, ticagrelor can increase adenosine levels by inhibiting adenosine reuptake and inducing adenosine triphosphate release from red blood cells, which stimulate vasodilation (34). These mechanisms of action of ticagrelor may result in better microvascular perfusion compared with clopidogrel.

Technically, CMD is diagnosed by assessing CFR and IMR (35). The speed of contrast movement during angiography also correlates with CMD (36). cTFC is a simple, reproducible, objective, and quantitative index of coronary flow (37). Although cTFC is a measure of epicardial flow, it depends on resistive components in the microvasculature. Studies have demonstrated that the cTFC value is a strong independent predictor of insufficient myocardial reperfusion after reopening the infarct-related artery (38). The MBG is used to evaluate myocardial perfusion by assessing the maximum intensity of contrast in the myocardium (39). Unsuccessful reperfusion (MBG = 0/1) correlated with a larger infarct size and higher cardiac mortality (40). One trial found that STEMI patients with a mortality of 24%, 10%, 6%, and 4% could be stratified with a MBG of 0, 1, 2, and 3 (*p *< 0.001), respectively (39).

In the present analysis, we found that loading with ticagrelor before PCI in STEMI patients reduced cTFC and improved MBG more significantly compared with clopidogrel. Sabbah et al. (41) found that loading with ticagrelor before primary PCI in STEMI patients was associated with a smaller infarct size and larger myocardial salvage index after a 3-month follow-up compared with clopidogrel. Another study found that a 180 mg ticagrelor loading dose might more effectively reduce microvascular injury, assessed by IMR, than a clopidogrel loading dose (42), which was consistent with our analysis. Moreover, Jeong et al. (43) investigated myocardial blood flow (MBF) using 13 N-ammonia positron emission tomography imaging in ACS patients who were treated with PCI. They found that MBF was higher after receiving ticagrelor for 6 months compared with clopidogrel. This may suggest that ticagrelor can improve coronary microcirculation function with short-term treatment before PCI or long-term maintenance treatment.

In conclusion, ticagrelor improved CMD more than clopidogrel in STEMI patients who underwent primary PCI.

### Limitations

The present meta-analysis has several limitations. First, we used trial-level data instead of individual patient data. Second, accurate details of the PCI procedures were absent from our analysis. Third, several RCTs included small populations. Fourth, significant heterogeneity still existed in the analysis of cTFC. However, because of the limited relevant data, no further analysis (e.g., subgroup analysis) could be conducted to reduce heterogeneity. Fifth, more novel indices (e.g., CFR, IMR, and hyperemic microvascular resistance) have been shown to more reliably evaluate microvascular dysfunction. However, there were only a few relevant clinical trials, possibly because of the additional financial burden and additional operational procedures that limit their use. Sixth, publication bias existed, which might influence the results. Seventh, the RCTs implemented various indicators of microcirculation. The use of more specific and accurate indicators should be encouraged.

## Conclusion

In conclusion, ticagrelor appeared to be superior to clopidogrel in improving coronary microvascular function in STEMI patients who underwent PCI. However, the RCTs that were analyzed herein had small sample sizes, and we detected various outcomes with regard to microcirculation. In the clinic, the choice between these two drugs should be made based on the most current research on clinical outcomes in patients with STEMI.
