# Supplementary material for: Ticagrelor vs. clopidogrel for coronary microvascular dysfunction in patients with STEMI: a meta-analysis of randomized controlled trials
Source: Front Cardiovasc Med. 2023 May 18;10:1102717. doi: 10.3389/fcvm.2023.1102717 (PMC10233131; doi:10.3389/fcvm.2023.1102717)
Supplement: Supplementary file 1 [file Table1.docx]

**SUPPLEMENTARY MATERIALS**

**Supplemental Table**

**Table 1.** Detailed search strategies.

**Table 2.** Risk of bias in included studies.

**Supplemental Figure**

**Figure 1.** Risk of bias assessment.

**Figure 2.** Funnel plot for cTFC.

**Figure 3.** Sensitivity analysis for cTFC.

**Figure 4.** Trim and fill analysis for MBG=3

**Table 1. Detailed search strategies.**

| **Database** | **Term** | **Result** |
| --- | --- | --- |
| PubMed | ("ticagrelor"[MeSH Terms] OR "ticagrelor"[Text Word]) AND ("Clopidogrel"[MeSH Terms] OR "Clopidogrel"[Text Word]) AND ("microcirculation"[MeSH Terms] OR "microcirculation"[Text Word] OR " microvascular "[MeSH Terms] OR "microvascular"[Text Word] OR " blood flow "[MeSH Terms] OR " blood flow"[Text Word] OR "coronary flow "[MeSH Terms] OR " coronary flow"[Text Word] OR "myocardial perfusion"[MeSH Terms] OR " myocardial perfusion "[Text Word] OR "coronary perfusion"[MeSH Terms] OR " coronary perfusion "[Text Word] OR "reperfusion"[MeSH Terms] OR "reperfusion "[Text Word] OR "coronary circulation"[MeSH Terms] OR " coronary circulation "[Text Word]) | n=97  citations |
| Embase | ('ticagrelor'/exp OR ticagrelor) AND ('clopidogrel'/exp OR clopidogrel) AND ('microcirculation'/exp OR microcirculation OR 'microvasculature'/exp OR microvasculature OR 'blood flow'/exp OR 'blood flow' OR 'coronary flow reserve'/exp OR (coronary AND flow AND reserve) OR 'fractional flow reserve'/exp OR 'fractional flow reserve' OR 'heart perfusion'/exp OR 'heart perfusion' OR microvascular OR (coronary AND flow) OR (myocardial AND perfusion)) | n=831 citations |
| Web of Science | ((((((((((TS=(microcirculation)) OR TS=(microvasculature)) OR TS=(blood flow)) OR TS=(coronary flow reserve)) OR TS=(fractional flow reserve)) OR TS=(heart perfusion)) OR TS=(microvascular)) OR TS=(coronary perfusion)) OR TS=(reperfusion)) OR TS=(coronary circulation)) AND (((TS=(ticagrelor)) AND TS=(Clopidogrel))) | n=387 |
| Cochrane Library | (MeSH descriptor: [Ticagrelor] explode all trees) AND (MeSH descriptor: [Clopidogrel] explode all trees) AND (((Microcirculation):ti,ab,kw OR (Coronary Circulation):ti,ab,kw OR (microvasculature):ti,ab,kw) OR (MeSH descriptor: [Reperfusion] explode all trees) OR (MeSH descriptor: [Coronary Circulation] explode all trees) OR (MeSH descriptor: [Fractional Flow Reserve, Myocardial] explode all trees) OR (("coronary flow reserve"):ti,ab,kw OR (coronary perfusion):ti,ab,kw OR (blood flow):ti,ab,kw OR (coronary flow):ti,ab,kw OR (myocardial perfusion):ti,ab,kw)) | n= 65 trials |

**Table 2. Risk of bias of included studies**

| **Study ID** | | **Random sequence generation**  **(Selection bias)** | **Allocation concealment (Selection bias)** | **Blinding of participants and personnel (performance bias)** | **Blinding of outcome assessment (Detection bias)** | **Incomplete outcome data (Attrition bias)** | **Selective reporting (reporting bias)** | **Other bias** |
| --- | --- | --- | --- | --- | --- | --- | --- | --- |
| **1** | **Winter 2014** | LOW | HIGH | Unclear | LOW | LOW | LOW | LOW |
|  | Assessment justification: | Using closed envelopes | Open label | Not specified | Blinded operators calculated angiographic corrected TIMI Frame count (cTFC) and myocardial blush grade (MBG). | No subjects were lost to follow-up. | Not specified | Not specified |
| **2** | **Mont'Alverne 2016** | Unclear | Unclear | Unclear | LOW | LOW | LOW | LOW |
|  | Assessment justification: | The patients were randomized | Not specified | Not specified | All angiograms were analyzed offline by two independent interventional cardiologists, blinded to clinical characteristics or the allocated treatment, for the  assessment of angiographic endpoints. | From the total 132 patients randomized, only one patient (angiography and primary angioplasty were not performed ultimately) was excluded from endpoint analysis. | Not specified | Not specified |
| **3** | **Li WH 2018** | Unclear | Unclear | Unclear | LOW | LOW | LOW | LOW |
|  | Assessment justification: | Randomly | Not specified | Not specified | The hemodynamics of the  coronary artery after emergency PCI was observed by three cardiologists who were blinded to the administration of the drugs. | No subjects were lost to follow-up. | Not specified | Not specified |
| **4** | **Wang X 2019** | Unclear | Unclear | Unclear | LOW | LOW | LOW | LOW |
|  | Assessment justification: | Randomly | Not specified | Not specified | After the procedure, two experienced operators blinded  to treatment assignment calculated TIMI flow grade,  corrected TIMI frame count (cTFC), and myocardial blush grade (MBG) in all patients. Disagreement was resolved by consensus. | No subjects were lost to follow-up. | Not specified | Not specified |
| **5** | **Cao B 2019** | LOW | Unclear | Unclear | Unclear | LOW | LOW | LOW |
|  | Assessment justification: | Random numbers | Not specified | Not specified | Not specified | No subjects were lost to follow-up. | Not specified | Not specified |
| **6** | **Liu Y 2019** | Unclear | Unclear | Unclear | Unclear | LOW | LOW | LOW |
|  | Assessment justification: | Randomly | Not specified | Not specified | Not specified | 210 patients were enrolled in this study, among which 2 cases were lost to follow-up. | Not specified | Not specified |
| **7** | **Hamilos 2021** | Unclear | HIGH | Unclear | LOW | HIGH | HIGH | HIHG |
|  | Assessment justification: | Randomization and administration  of the P2Y12 inhibitor took place | Open label | Not specified | Angiograms were blindly evaluated in an independent core lab (Cardialysis, Rotterdam, the Netherlands) and pre- and post PCI TIMI myocardial perfusion grade (MPG), corrected TIMI  frame count (CTFC). | From the 259 patients who underwent PCI, 154 angiograms were  analysable for the primary endpoint (60.5%). | 259 patients underwent PCI and only 154 patients analysable for the primary endpoint(60.5). | In all patients enrolled, thrombolysis with a fibrin-specific agent constituted the reperfusion modality of choice, since primary PCI was not feasible within two hours from first medical contact. |

**Figure 1.** Risk of bias assessment

**
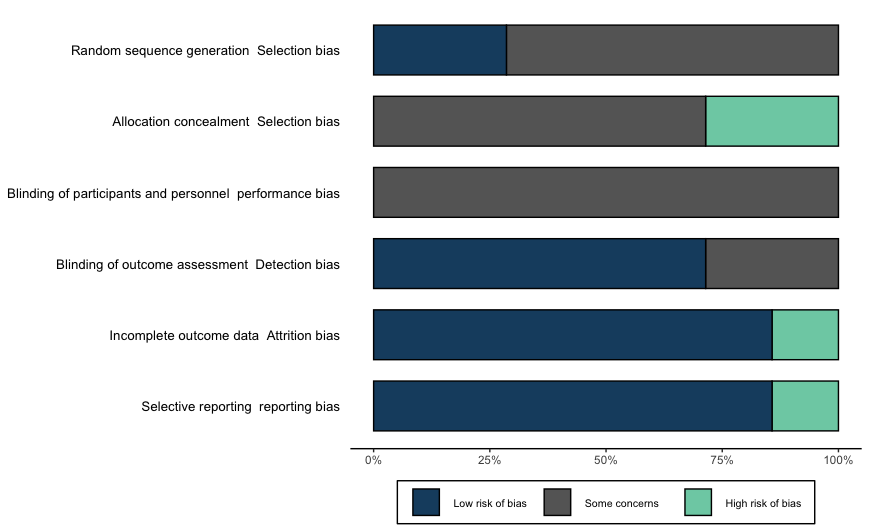
**

**
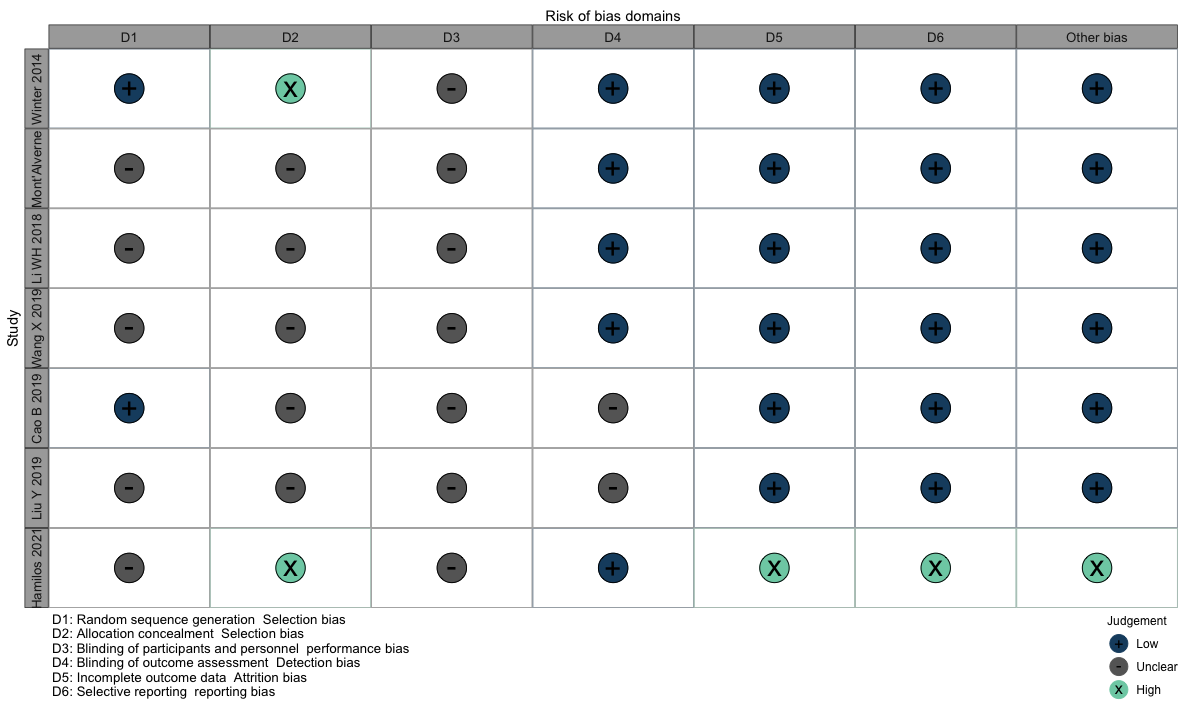
**

The risk of bias in included studies were summarized in the graph. All studies were assessed at a low to moderate risk of bias using the Cochrane risk-of-bias tool.

**Figure 2.** Funnel plot for cTFC


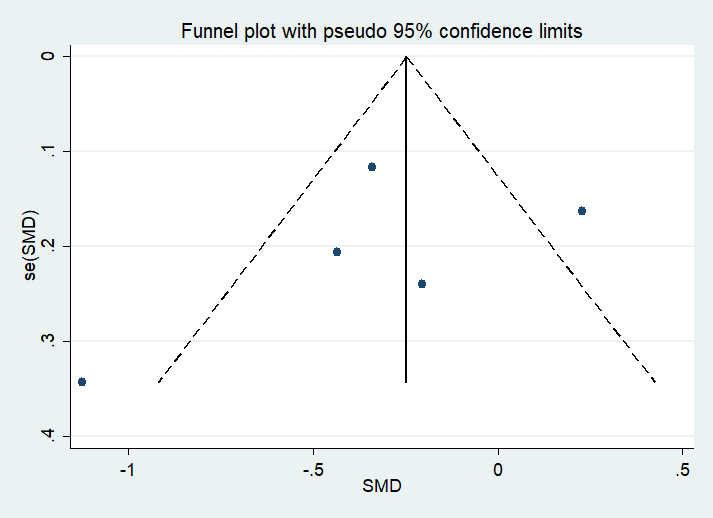


**Figure 3.** Sensitivity analysis for cTFC


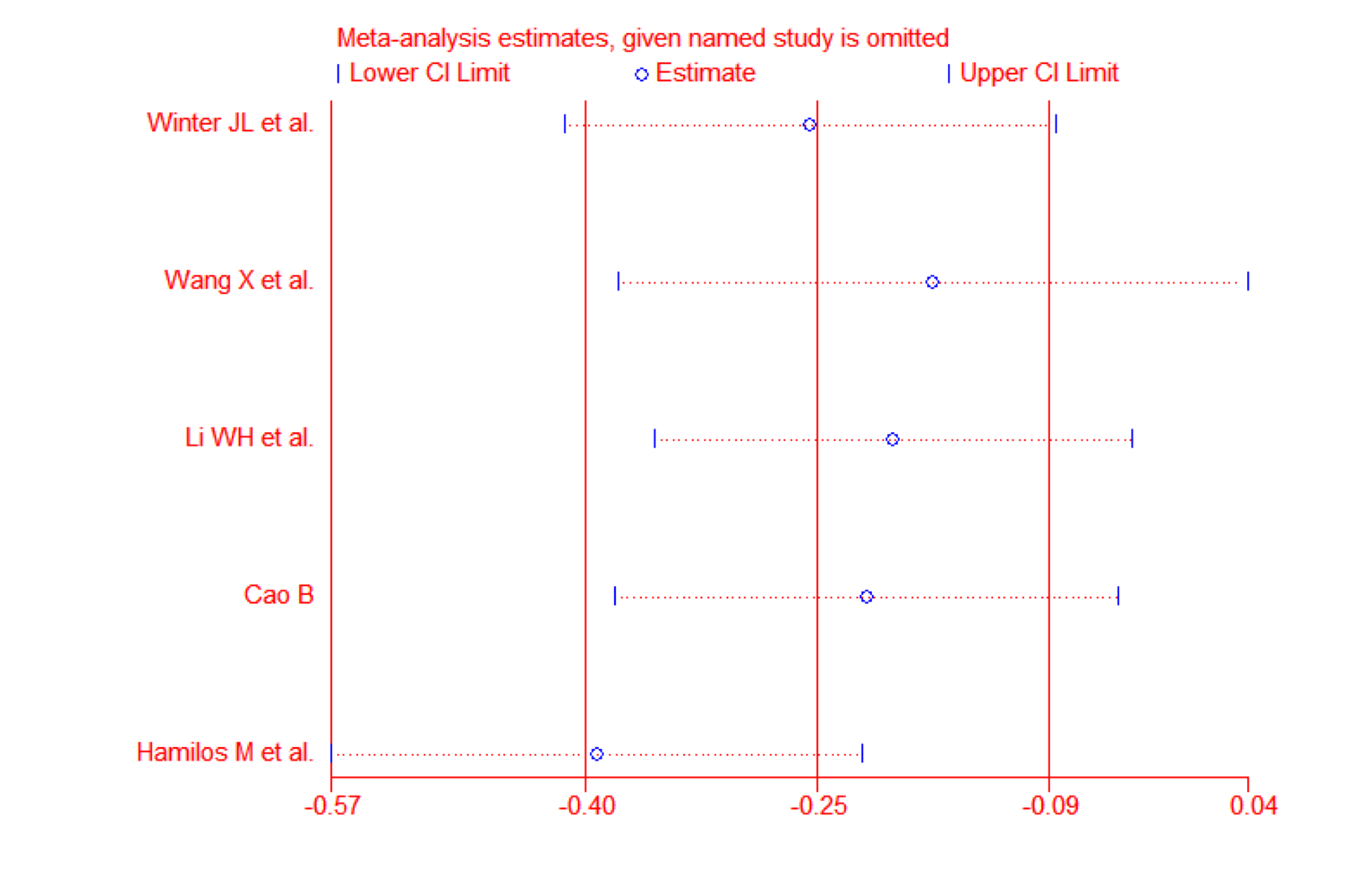


The sensitivity analysis for cTFC indicated significant heterogeneity in one study.

**Figure 4.** Trim and fill analysis for MBG=3


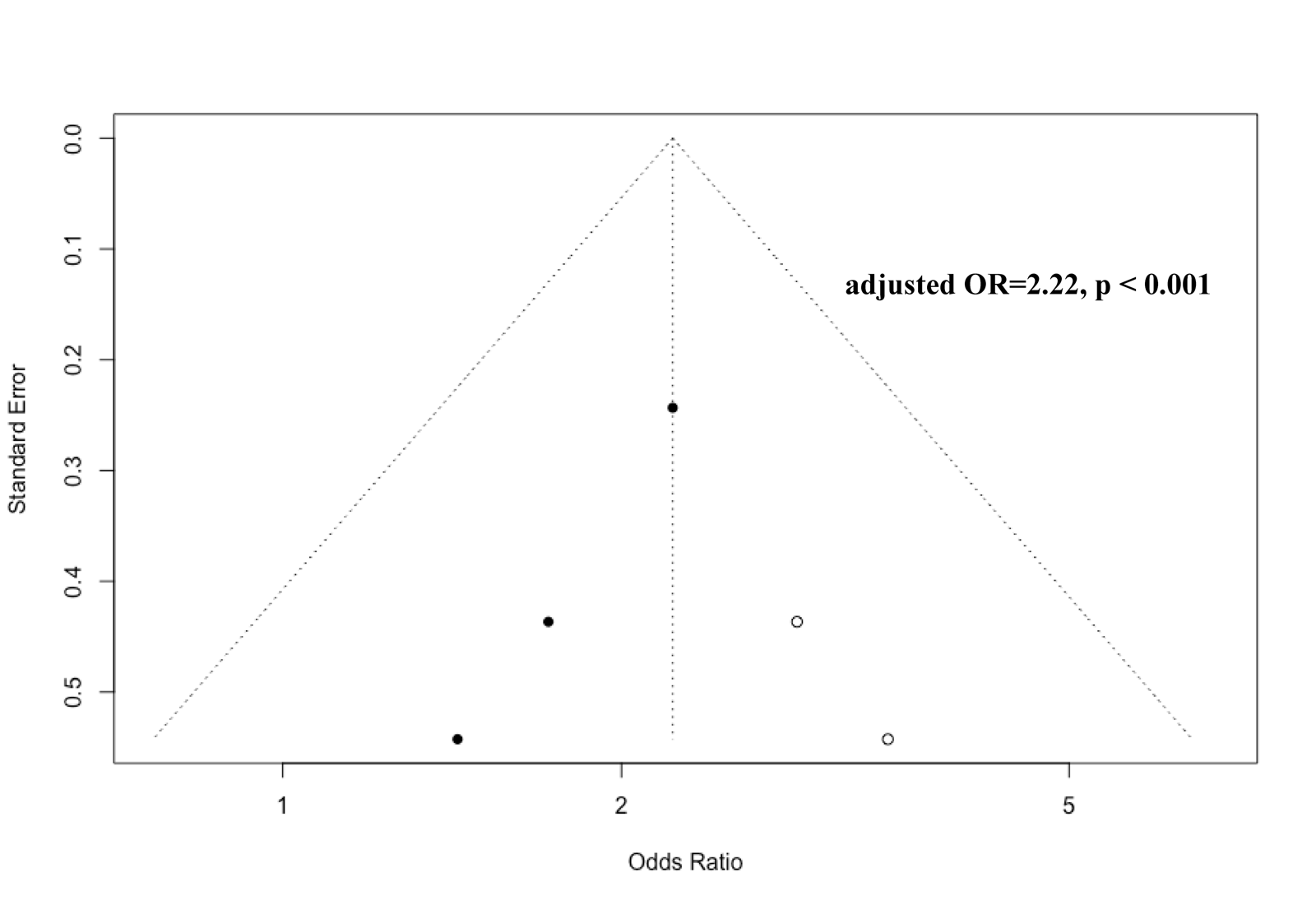


Adjusted pooled estimate with trim and fill analysis for MBG=3
